# Introduction of Multidisciplinary Prescribing Team Meeting in Dragon Square North Staffordshire Child and Adolescent Mental Health Services (CAMHS) as a Pilot and Analysis of Its Overall Impact, Including Waiting Times

**DOI:** 10.1192/bjo.2025.10431

**Published:** 2025-06-20

**Authors:** Syed Shazia Rizvi, Bindu Poornamodan, Sadia Shafique

**Affiliations:** 1 North Staffordshire Combined Healthcare Trust, Stoke on Trent, United Kingdom; 2 North Staffordshite Combined Healthcare Trust, Stoke on Trent, United Kingdom; 3 North Staffordshire Combined Healthcare Trust, Stoke on Trent, United Kingdom

## Abstract

**Aims:** Once referred to child and adolescent mental health services (CAMHS), children and young people often report long waiting times for assessment, diagnosis, and treatment (Young Minds, 2022). The COVID-19 pandemic also brought an unprecedented mental health crisis (UNICEF, 2021), increasing the burden on over-stretched CAMHS, thereby increasing the waiting time. Most research and quality improvement projects on ‘waiting lists’ focusses on how to avoid missed appointments or effectively manage booking/triage systems. This Multidisciplinary Prescribing Meeting Pilot project was initiated in an attempt to reduce the waiting times.

Objective: To assess the impact of introducing fortnightly Multi-Disciplinary Prescriber meeting in Dragon Square.

**Methods:** This Pilot programme ran from November 2022 to August 2023. Referrals to the Prescribing team came from weekly Child and Adolescent Mental Health Services (CAMHS) Multi-Disciplinary team meetings. These patients were discussed by prescribers comprising a Consultant psychiatrist, Core psychiatry trainee, Higher psychiatry trainee, a Staff grade doctor, Nurse Consultant and Non-medical prescribers. After discussion patients were allocated to prescribers based on the complexity of cases. Prior to this non-medical prescribers were mainly reviewing children with Attention Deficit Hyperactivity Disorder. Complex cases were brought back for discussion when prescribers needed support. There was also an opportunity to step cases up or down based on their complexity and the level of support required.

**Results:** Waiting times have significantly reduced during the Pilot period. The average wait time reduced from 59.33 days to 24.5 days.

The highest wait time before the Pilot was 86 days which reduced to 42 days. Similarly, the lowest wait time before pilot was 50 days which reduced to 11 days.

This meeting also provided peer supervision for the prescribers.

Analysis of the data showed a positive impact in multiple ways. The Strengths of the Pilot included a reduction in waiting times, complexity Based Patient Assignment, peer supervision and Learning and upskilling of Prescribers. The team did not identify any weaknesses except for the time commitment.

**Conclusion:** This project had a significant positive impact on the service overall.

This model can be successfully implemented in other teams with a strong cohort of prescribers.

